# A Rare Presentation of Congenital Chilaiditi Syndrome: Symptomatic Hepatobowel Entrapment

**DOI:** 10.7759/cureus.33714

**Published:** 2023-01-12

**Authors:** Waqar Akram, Lavi Singh, Sanket Shah, Shaheer Hussain, Vishrut Shah, Aaiz Hussain, Hasnan M Ijaz, Mustafa Rahim

**Affiliations:** 1 Internal Medicine, Raleigh General Hospital, Beckley, USA; 2 College of Liberal Arts and Science, Wayne State University, Detroit, USA; 3 Internal Medicine, Westside Regional Medical Center, Plantation, USA; 4 Chicago College of Osteopathic Medicine, Midwestern University, Downers Grove, USA; 5 Dr. Kiran C. Patel College of Allopathic Medicine, Nova Southeastern University, Davie, USA

**Keywords:** pneumoperitoneum, ct (computed tomography) imaging, hepatobowel entrapment, chilaiditi sign, chilaiditi

## Abstract

The displacement and trapping of the colon between the liver and the right hemidiaphragm are known as the Chilaiditi sign or syndrome. The Chilaiditi sign presents in an asymptomatic patient, while Chilaiditi syndrome presents with symptoms such as abdominal pain, distension, and constipation, in addition to complications such as perforation, volvulus, and bowel obstruction. It is often misdiagnosed as pneumoperitoneum or free air under the diaphragm and liver, often seen on the abdomen and chest radiography. It more commonly presents in males than in females. Here, we present the case of a 37-year-old female who reported abdominal pain and persistent constipation. An abdominal CT scan showed entrapment of a bowel segment, which is referred to as the Chilaiditi sign. The patient’s presentation with hepatobowel entrapment and persistent gastrointestinal symptoms was diagnosed as Chilaiditi syndrome. This presentation entails a conservative management approach. The aim of this report is to educate about the rare occurrence of Chilaiditi sign and Chilaiditi syndrome as a differential diagnosis to often misdiagnosed critical conditions such as pneumoperitoneum and intestinal perforation. Correctly identifying these patients will reduce overtreatment and help improve outcomes.

## Introduction

Chilaiditi sign has an estimated incidence of 0.025% to 0.28% worldwide. The incidental radiographic impression of gas accumulation under the right hemidiaphragm caused by displacement and entrapment of the colon (typically transverse) between the liver and right diaphragm, without causing symptoms, is known as the Chilaiditi sign. Patients presenting with symptoms such as abdominal pain, abdominal distention, and constipation due to this colonic interposition are referred to as having Chilaiditi syndrome [1]. Although no specific genetic condition is directly involved with the presence of a Chilaiditi sign, it has been seen in patients with multiple endocrine neoplasia type 2b and myotonic dystrophy type 1 [2]. This report educates about the rare occurrence of Chilaiditi sign and Chilaiditi syndrome as a differential diagnosis to often misdiagnosed critical conditions, such as pneumoperitoneum and intestinal perforation, that necessitate an aggressive treatment approach. Correctly identifying patients with Chilaiditi syndrome will reduce overtreatment and help improve outcomes.

## Case presentation

**Figure 1:**
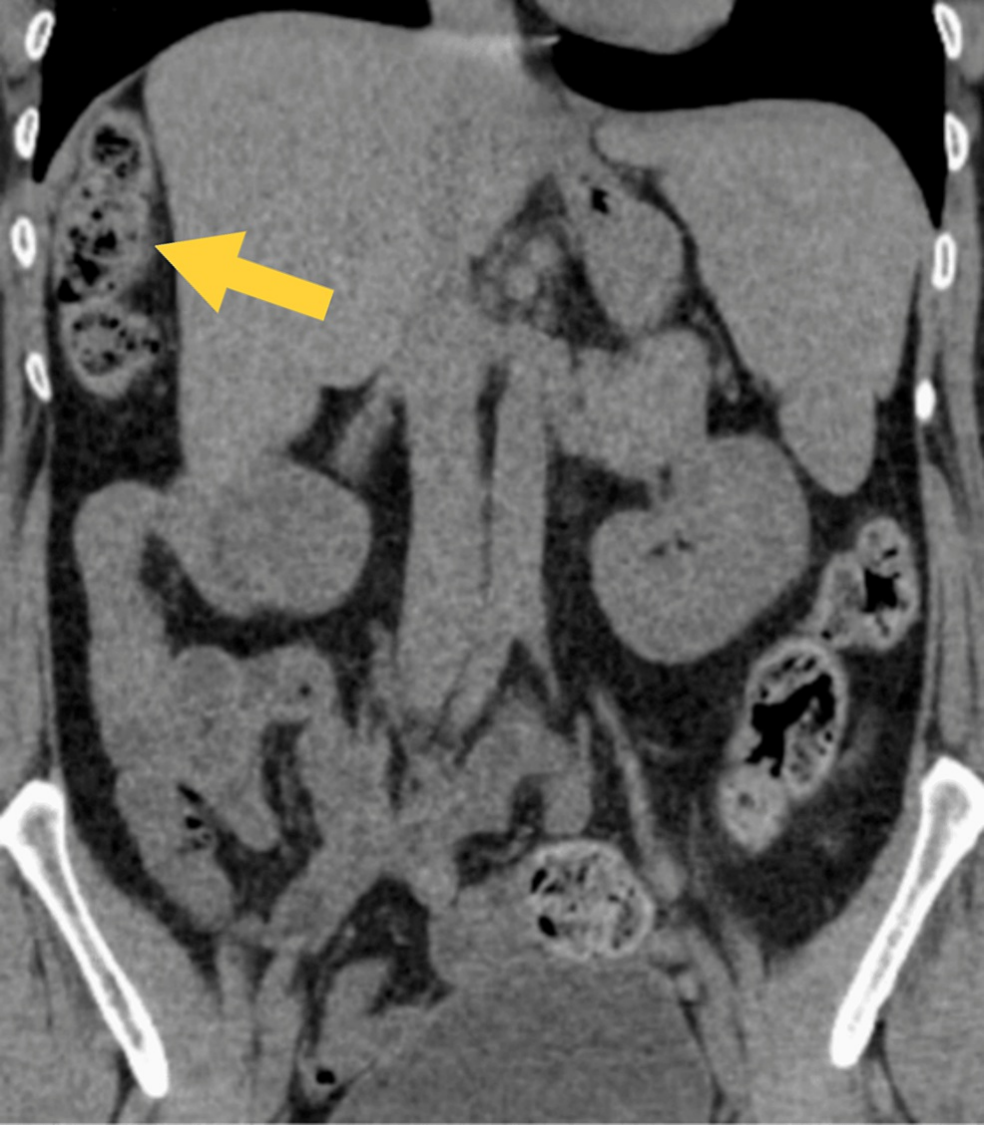
Subdiaphragmatic entrapment of a large bowel loop behind the right edge of the liver (yellow arrow).

**Figure 2:**
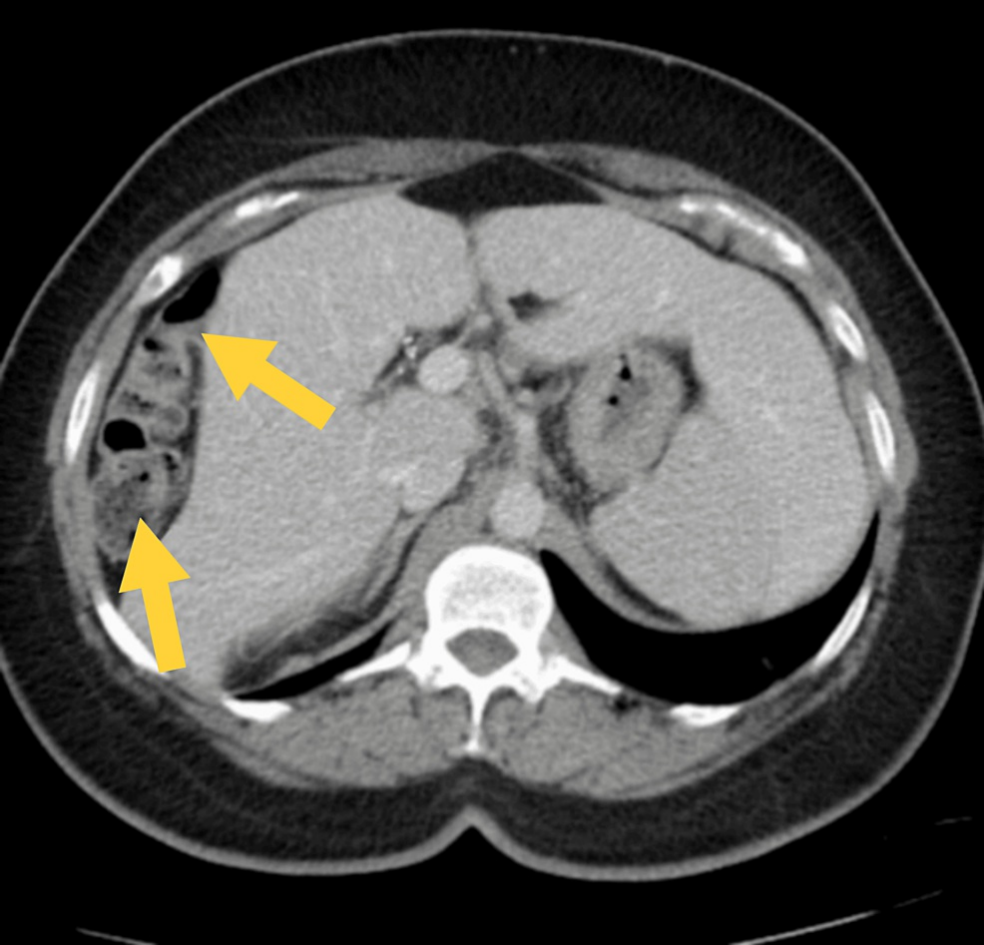
A segment of the large bowel (bottom arrow) looped under the diaphragm behind the right hepatic border (top arrow).

A 37-year-old female presented to the emergency room on multiple occasions with constipation and diffuse, sharp, stabbing abdominal pain with nausea, dry heaving, and occasional vomiting with biliary tinge content. Her medical history was significant for acute pancreatitis, hepatitis C infection, and malrotation of the intestine. She reported having one to two bowel movements per week for many years. She occasionally had hematochezia and persistent constipation. Complete blood count (Table 1) showed an elevated white blood cell and neutrophil count. Liver function tests (Table 2) were within normal limits. Abdominal CT scans (Figures 1, 2) showed a large bowel segment entrapped between the right liver margin and diaphragm suggestive of the Chilaiditi sign. Our patient had a positive outcome with conservative management.

**Table 1:** Patient’s white blood cell count.

Laboratory tests	Value	Normal range
Total white blood cells	12.46 H	3.98–10.04 × 10^3^/µL
Absolute neutrophils	9.27 H	1.56–6.13 × 10^3^/µL
Absolute lymphocytes	2.30	1.18–3.74 × 10^3^/µL

**Table 2:** Patient’s liver function test.

Laboratory tests	Value	Normal range
Total bilirubin	0.41	0.20–1.00 mg/dL
Aspartate transaminase	23	15–37 U/L
Alkaline phosphatase	58	46–116 U/L
Albumin	3.6	3.4–5.0 g/dL

## Discussion

An incidental radiological finding of displacement and trapping of the bowel between the liver and right hemidiaphragm must meet specific criteria to be diagnosed as pseudo-pneumoperitoneum, also referred to as the Chilaiditi sign. Pseudo-pneumoperitoneum is diagnosed by meeting the following three criteria: the right hemidiaphragm must be elevated above the liver by intestines, the bowel must be distended by air, and the superior margin of the liver must be depressed below the level of the left hemidiaphragm [1]. The Chilaiditi sign typically presents asymptomatically; however, if the patient is experiencing symptoms due to this bowel interposition, this condition is referred to as Chilaiditi syndrome. It presents with symptoms such as abdominal pain, distention, and constipation, or complications such as perforation, volvulus, and bowel obstruction. Although there is no genetic association with this syndrome, there has been a documented co-incidence of Chilaiditi syndrome with multiple endocrine neoplasia type 2b and myotonic dystrophy type 1 [2,3].

Abnormal anatomy of suspensory ligaments of the transverse colon or falciform ligament allows the bowel to interpose itself between the liver and hemidiaphragm, giving the impression of trapped air pneumoperitoneum on radiographs or ultrasound. The key differentiating factor between pneumoperitoneum and pseudo-pneumoperitoneum is that changing the patient’s position does not affect the radiolucency on the radiograph or the gas echo on ultrasound in the case of a pseudo-pneumoperitoneum [4,5]. Patients presenting with abdominal pain related to Chilaiditi syndrome should be approached with multiple differential diagnoses in mind. Additional diagnoses to consider in a patient with Chilaiditi sign are pneumoperitoneum and subphrenic abscess. These more emergent diagnoses can be ruled out by noting the presence of plicae circulares or haustral markings of the transverse colon under the diaphragm on radiography [4]. In a clinically stable patient, a CT scan is the most specific test to distinguish the presence of air under the diaphragm or within the lumen of a transverse colon [6]. Colonoscopy should be performed with utmost caution as passing the instrument in the angulated portions of the trapped bowel can cause additional air to accumulate, thus increasing the risk of perforation [7]. It is essential to distinguish Chilaiditi syndrome from other diagnoses as it requires a conservative approach for management.

A study measuring the length of the colon in men and women found that the total colonic length is greater in women than in men, with the most noticeable difference in length in the transverse colon [8]. Although this anatomical variation may support the presumption that greater length and laxity of the transverse colon provide a favorable circumstance for bowel interposition, it does not negate that the Chilaiditi sign has a higher incidence in men than in women by a ratio of 4:1 [9]. Typical anatomical variations that lead to this disorder are the complete absence of the colonic suspensory ligaments and hepatic falciform ligament, redundant colon, paralyzed right hemidiaphragm, or enlarged thorax allowing space for the colonic interposition [10].

Although Chilaiditi sign is a benign incidental finding, in symptomatic patients Chilaiditi syndrome has two approaches to management, namely, conservative treatment and surgical intervention. The conservative approach includes fluid supplements, peristalsis stimulation, enema, nasogastric decompression, and fasting [4]. A case reported by Weng et al. described the conservative management of a patient presenting with Chilaiditi syndrome who was confirmed negative for Chilaiditi sign on repeat CT scans six days later. Following treatment, the patient was clinically stable and could pass a stool while tolerating a liquid diet [11]. Surgical options for management in symptomatic patients who failed conservative management include colectomy and colopexy [12]. Garcia and Ashurst reported a successful outcome with da Vinci’s robotic surgical intervention in a patient presenting with persistent abdominal pain and diarrhea associated with Chilaiditi syndrome [1].

## Conclusions

This case describes a patient with a radiological finding of bowel entrapment and displacement between the liver and right hemidiaphragm. The patient was treated conservatively for Chilaiditi syndrome with a positive outcome. The Chilaiditi sign often gets misinterpreted as pneumoperitoneum and can lead to overtreatment. Although rare, it is crucial to keep the Chilaiditi sign in mind as a differential diagnosis when a suspected pneumoperitoneum is incidentally observed on radiography. Furthermore, in symptomatic patients with abdominal and gastrointestinal symptoms, Chilaiditi syndrome should be considered alongside severe etiologies such as intestinal perforations and obstruction because Chilaiditi Syndrome necessitates a conservative treatment approach. Correctly identifying these patients will reduce overtreatment and help improve outcomes.
